# Supplementary material for: Are carbon emissions trading and green financial instruments synergistic? -Comprehensive quantitative research based on content analysis
Source: PLoS One. 2024 Mar 7;19(3):e0298601. doi: 10.1371/journal.pone.0298601 (PMC10919601; doi:10.1371/journal.pone.0298601)
Supplement: S1 Data — (ZIP) [file pone.0298601.s001.zip › Data/地级市Z_F工作报告.zip!/▒▒╛⌐/└┤╫╘╣1⁄2╓┌║┼í╛┬φ┐╦╩2╛▌═°í┐_2003.docx]

——2003年1月13日在北京市第十二届人民代表大会第一次会议上

北京市市长　刘淇

各位代表：

　　现在，我代表北京市人民政府向大会作工作报告，请予审议，并请市政协各位委员提出意见。

　　一、五年工作的回顾

　　北京市第十一届人民代表大会第一次会议以来，全市各族人民在中共北京市委领导下，坚持以邓小平理论和“三个代表”重要思想为指导，坚决贯彻中央重大决策和对北京工作的一系列指示精神，坚持大力发展首都经济的正确方向，抓住机遇，开拓创新，加快发展，圆满完成了“九五”计划，提前实现了现代化建设的第二步战略目标，取得了“十五”计划良好开局，向率先基本实现现代化的目标又迈进了一步。

　　经济发展步入新阶段，综合实力迈上新台阶。认真贯彻扩大内需的方针，克服世界经济波动带来的不利影响，国民经济持续快速健康发展，经济总体实力显著增强，宏观经济效益不断改善。五年来，国内生产总值年均增长10.5%，2002年，实现3130亿元，同比增长10.2%，人均国内生产总值达到3355美元。五年累计完成全社会固定资产投资6960亿元，实现社会消费品零售额7290.1亿元，年均分别增长12%和10.7%。地方财政收入由1997年的182.3亿元增加到534亿元，年均增长25.7%。社会发展水平总指数和信息化水平总指数均居全国首位。经济结构调整成效显著，三次产业发展确立新格局，结构优化对经济增长的拉动效应日益突出。中关村科技园区“三年大变样”的目标全面实现，2002年增加值、上缴税金和出口创汇分别完成537亿元、110亿元和31亿美元，分别比三年前增长1.4倍、1.7倍和2.2倍。全市高新技术产业增加值占工业增加值的比重达到28.9%。振兴现代制造业已经起步。现代都市农业迅速发展，农村城市化进程加速推进。现代服务业蓬勃兴起，新的增长点不断涌现，第三产业增加值占国内生产总值的比重由1997年的54.5%提高到61.3%。首都经济现代化步伐明显加快、增长方式开始发生重大转变，抗风险能力和综合竞争力得到较大幅度的提升。

　　社会主义市场经济体制初步建立，对外开放水平进一步提高。国有企业改革脱困三年目标如期实现，公有制经济进一步壮大，私营个体特别是民营高科技企业等非公有制经济加快发展。政府机构改革取得阶段性成果，政府职能转变步伐加快。市政府部门由67个减少到54个，内设机构减少118个，行政编制精简50%。行政审批制度改革稳步推进，市级审批事项共精减587项，精减比例达45%。公共财政框架体系初步构建，市与区县财政分税体制为经济发展注入了新的活力。市场体系建设全面展开，经济秩序得到改善。社会保障体系框架基本形成。投融资体制改革持续深化，基础设施投资补偿机制逐步完善，融资渠道不断拓宽。经营性土地供应进入市场化运作。体制和机制创新对经济社会发展的促进作用更加明显。投资贸易环境显著改善，利用外资的观念、模式和领域实现新突破，五年实际利用外资累计达到103.1亿美元，是上五年的1.5倍；地方外贸出口累计实现213.9亿美元，是上五年的2.1倍。对外交往日益活跃，成功举办了一系列大型国际活动。年接待入境旅游者突破300万人次。对外开放朝着全方位、多层次、宽领域的方向深入发展。

　　城市建设日新月异，现代化国际大都市面貌初步显现。始终坚持基础设施优先发展，五年累计完成基础设施投资超过1700亿元，是上五年的2.1倍。四环路、广安大街等相继通车，城区路网加密步伐加快，全市道路总长度达到4400公里，高速公路总长度达到463公里。复八线、城市铁路西线(十三号线)投入运营，轨道交通运营里程达到74公里。水、电、气、热、通信等基础设施的供应能力大幅度提高。商务中心区等城市功能区和重点商业街区建设步伐加快，一批文化设施和大型标志性建筑建成。城市布局继续完善，承载能力和服务功能迈上新台阶。制定了《历史文化名城保护规划》，健全了历史文化名城整体保护、历史文化保护区和文物保护单位三个层次的保护体系，实施“3.3亿元文物抢修计划”，带动相关资金的投入，圆明园、明城墙遗址、菖蒲河等文物抢险修缮和腾退整治取得重大突破，古都风貌得到有效保护。开展了大规模的环境污染治理和生态建设。自1998年以来，政府和社会各界共投入466亿元，实施了八个阶段的大气污染控制措施，2002年空气质量二级和好于二级的天数占全年的55.6%，比1998年提高了28.2个百分点。城市污水处理率为45%，垃圾无害化处理率86.5%，分别比1997年提高了23和47.5个百分点。城区建成51处集中大绿地，市区绿化隔离带建设三年时间实现十年绿化目标，绿化面积达到110平方公里，环绕京城的三道绿色生态屏障基本形成，全市林木覆盖率和城市绿化覆盖率分别达到44.5%和40.2%，生态环境显著改善。以街道、社区建设为重点，不断深化城市管理体制改革，推行城市管理重心下移。组建城管监察队伍，城市管理综合执法得到加强。城市规划、建设和管理的水平进一步提高，适应现代化大都市发展需要的城市管理新格局初步确立。

　　大力发展社会主义民主，确保首都社会的安全稳定。坚决执行人大及其常委会决议，政府组成人员向人大常委会述职，自觉接受人大监督。加强与人民政协联系，完善与人民群众联系渠道，提高了决策民主化、科学化水平。五年共办复全国和市人大代表议案、建议以及政协委员提案15330件。加强村民自治、社区自治组织建设，政务、村务、厂务公开逐步规范化、制度化，扩大了基层民主。民族、宗教、侨务和对台工作取得新进展。坚持依法治市，切实推进政府法制建设，五年提请市人大常委会审议通过地方性法规和法规性决定68项，制定政府规章210项，依法行政水平不断提高。廉政建设进一步加强。“三五”普法教育取得明显成绩，“四五”普法教育进展顺利，法律服务体系日益健全，公民法律意识得到加强，为首都各项事业发展创造了良好的法律氛围。加强社会治安综合治理，依法严厉打击各种严重犯罪活动，社会治安形势保持稳定。大力实施科技强警战略，提高反恐防暴能力，首都公安警务保障迈上了新台阶。与“法轮功”邪教组织的斗争取得决定性胜利。建立维护首都安全稳定的长效机制，深入开展人民内部矛盾纠纷排查调处工作，有效遏制了重大安全事故的发生，确保了首都政治稳定和社会安定。

　　各项社会事业全面进步，精神文明建设成绩显著。“首都二四八重大创新工程”取得重要成果，区域创新体系框架基本形成，科技持续创新能力明显增强。科技体制改革进一步深化，与中央在京科技力量的合作更加广泛深入，市属科研院所转制工作基本完成。积极推进教育改革和布局调整，全面实施素质教育，基础教育向高标准、高质量的目标迈进，率先普及了高中阶段教育，实现了高等教育大众化。五年共投入教育经费306.2亿元，办学条件明显改善。高校后勤社会化改革稳步推进，大学生公寓建设三年竣工83万平方米。文化事业繁荣活跃，五年共有42部作品获全国“五个一工程”奖。文化产业初具规模。群众文化生活更加丰富多彩。卫生、体育和计划生育事业都有长足发展，城乡居民健康水平明显提高。在广大干部群众中深入进行邓小平理论和“三个代表”重要思想的学习教育，“三讲”集中教育取得明显成效。认真贯彻《公民道德建设实施纲要》，开展一系列群众性精神文明创建活动，六个区县、三个行业先后被评为全国文明区县和文明行业，涌现出一大批文明社区、文明村镇和文明单位。广泛开展“双拥”共建活动，国防教育、征兵工作和国防后备力量建设不断加强，优抚安置政策得到落实，驻京部队在首都现代化建设中发挥了重要作用。

　　城乡居民收入大幅增加，人民生活质量明显改善。2002年，城镇居民人均可支配收入12453.3元，农民人均纯收入5880.1元，分别比1997年增长59.4%和56.3%。农民增收致富和扶贫开发成效显著，人均纯收入1500元以下的低收入村全部消除，边远山区农民人均纯收入比1997年翻了一番。居民消费层次逐步升级。食品放心工程进展顺利。2000年以来加快了危旧房改造步伐，三年共拆除危旧房409万平方米，相当于前十年的总和。加大了住宅建设力度，城镇居民人均住房使用面积达到18平方米。高度重视并做好就业工作，五年新增64.8万个就业岗位，安置30万下岗职工，2002年城镇登记失业率为1.35%。困难群众的基本生活得到保障。坚持每年办60件实事，人民群众越来越感到方便和舒心。人民生活提前实现了由温饱到小康的历史性跨越。

　　五年来，我们圆满完成了新中国成立50周年庆典和党的十六大服务保障工作等一系列重大政治任务。百年奥运，中华圆梦，赢得2008年奥运会举办权，激发了全市和全国人民的爱国热情。这一切转化为首都改革开放和现代化建设的强大精神动力，全市上下呈现出“人心齐、抓机遇、求发展”的可喜局面。

　　可以说，过去的五年，是首都经济取得突破性进展，综合经济实力大幅度跃升，人民群众得到实惠最多的五年；是城市面貌日新月异，服务功能日益增强，现代化水平不断提高的五年；也是各项社会事业全面发展，社会主义精神文明和民主法制建设不断取得新进步的五年。这一切成绩的取得，离不开党中央、国务院的亲切关怀和正确领导，离不开全国方方面面的大力支持和帮助，是全市各族人民辛勤劳动和团结奋斗的结果。在这里，我代表市政府，向全市各族人民，向人大代表、政协委员，向各民主党派、各人民团体、社会各界人士，向中央在京单位、人民解放军和武警部队以及各兄弟省、区、市，向所有关心支持首都建设的香港特别行政区同胞、澳门特别行政区同胞、台湾同胞、海外侨胞和国际友人，表示崇高的敬意和衷心的感谢！

　　在充分肯定成绩的同时，必须清醒地看到，在全市经济和社会发展过程中仍存在着不少矛盾和问题。主要是经济发展水平与建设现代化国际大都市的目标有很大差距，观念、体制、机制和科技创新的任务还很艰巨。部分地区发展水平相对落后。工业结构不合理的矛盾比较突出，支撑经济发展的产业基础还不牢固。社会保障体系还不健全，部分群众生活比较困难，就业压力很大。交通拥堵没有得到有效缓解，资源保护、环境治理与生态建设任务繁重，城市管理的长效机制有待巩固和完善。城市文明程度与首都的地位仍不相适应。社会不稳定因素依然存在。政府机关及公务员的工作作风、服务质量有待进一步改进和提高。我们要高度重视这些问题，并采取有效措施切实加以解决。

　　过去五年的实践，进一步深化了我们对首都现代化建设规律的认识，积累了许多宝贵的经验。

　　第一，始终坚持以经济建设为中心，聚精会神搞建设，一心一意谋发展。这是实践“三个代表”重要思想的根本途径。必须从首都的性质地位出发，立足于发挥北京的比较优势，着眼于我国和世界科技进步、结构调整的大趋势，整合首都生产力资源，大力实施首都经济发展战略，不断深化认识、丰富内涵，走出一条具有时代特征、中国特色、首都特点的发展路子，努力为先进生产力发展开辟道路，推动全市经济实力和综合竞争力不断迈上新台阶。

　　第二，始终坚持全心全意为人民服务的宗旨，立党为公、执政为民。实现人民生活的富裕幸福，是我们一切工作的根本出发点和归宿。必须时刻把人民的利益放在心中，凡是涉及群众利益的事情，都应当尽心尽力尽快办好。要深入群众开展调查研究，勤政廉洁，艰苦奋斗，为人民群众多办实事，认真解决好就业、出行、住房、环境、安全等与人民群众利益直接相关的热点、难点问题，尽力使人民群众在改革与发展中得到更多实惠。

　　第三，始终坚持抓住机遇、把握大局，调动一切积极因素，实现跨越式发展。抓住机遇，是加快首都发展的必然要求。必须牢固树立机遇意识，紧紧抓住一切有利于加快发展的大好时机，用足用好机遇带来的发展条件，创造性地开展工作。做好“四个服务”是首都工作的大局。必须竭尽全力履行“四个服务”的职责，积极争取中央各部门和在京单位对北京工作的指导和帮助。加强与各省区市的联系与合作，凝聚和调动各方面的积极性，实现首都建设与发展的新飞跃。

　　第四，始终坚持改革开放，维护首都稳定，为首都现代化建设提供强大动力和坚实保障。必须与时俱进、开拓创新，坚持社会主义市场经济改革方向，消除束缚生产力发展的体制障碍，适应经济全球化和我国加入世贸组织的新形势，在更大范围、更广领域、更高层次上参与国际经济合作与竞争。要把转变政府职能和提高办事效率作为改革的重要环节，不断改进政府工作作风，不断提高管理效能和服务水平，不断为经济增长、城市发展和人民群众安居乐业创造良好的社会环境。要把维护首都安全稳定作为第一位的政治任务，正确处理改革发展稳定的关系，把改革的力度、发展的速度和社会的可承受程度有机统一起来，在社会稳定中推进改革与发展，在改革发展中促进社会稳定，夺取首都改革开放和现代化建设的新胜利。

　　第五，始终坚持科教兴国和可持续发展战略，促进经济社会发展与人口资源环境相协调。必须充分发挥科学技术作为第一生产力的决定性作用，以及教育的先导性、全局性、基础性作用，加强科技教育同经济的结合，加快科技成果向现实生产力转化。环境也是生产力。必须把控制人口、节约资源、保护环境摆在十分突出的位置，大力推进环境污染治理和生态建设，全面加强水、土地等资源管理和利用，不断提高首都可持续发展能力，向空气清新、资源节约、环境优美、生态良好、人居和谐的现代文明城市的目标迈进。

　　第六，始终坚持大力发展先进文化，推动物质文明和精神文明协调发展。大力推进民主法制建设，切实加强精神文明建设，是社会主义现代化建设的重要内容和保证。必须坚持依法治国与以德治国相结合，以“建首善、创一流”为目标，“两手抓、两手都要硬”，在抓好物质文明建设的同时，大力发展先进文化，不断丰富人们的精神世界，提高市民的文明素质和城市的文明程度，为首都经济社会发展提供强大的精神动力和智力支持，使广大人民在建设现代化国际大都市的征程上，始终保持奋发有为、昂扬向上的精神风貌。

　　二、今后五年的主要任务和2003年工作的建议

　　党的十六大确立了全面建设小康社会的奋斗目标，指出有条件的地方可以发展得更快一些，率先基本实现现代化，明确要求努力办好2008年奥运会，这是对北京极大的鼓舞和鞭策。我们要全面贯彻十六大精神，认真落实市第九次党代会的部署，全面建设小康社会，为实现“新北京、新奥运”的构想，为在2008年人均国内生产总值达到6000美元，率先基本实现现代化而努力奋斗。

　　面对这样重大的历史任务，必须把发展作为第一要务，抓住机遇，加快发展。我国正处在一个重要战略机遇期。今后五年，首都发展具备许多有利条件，特别是举办奥运会，就是一个十分难得并且可以大有作为的历史性机遇。机遇稍纵即逝，必须及时抓，才能取得主动权，赢得发展优势；机遇只是发展的良好时机和条件，必须抓紧抓实，才能转化为发展的实际成果。我们一定要牢固树立强烈的机遇意识，用足用好机遇，充分发挥主观能动性，去争取最大的效果。

　　面对这样重大的历史任务，我们必须突出抓好优化发展环境，特别是投资和消费环境，大力改善首都的政策、法制、服务和舆论等环境，尤其要注重制度建设和创新，坚决纠正一切妨碍发展的认识和做法，坚决废除一切束缚发展的政策和规定，坚决革除一切影响发展的体制障碍和机制弊端，进一步调动一切积极因素，真正实现一切劳动、知识、技术、管理和资本的活力竞相迸发，一切创造社会财富的源泉充分涌流，造福于人民，贡献于现代化。

　　今后五年，政府工作的总体要求是：以邓小平理论和“三个代表”重要思想为指导，全面贯彻党的十六大精神，认真落实中央的一系列重大决策和部署，继续落实市第九次党代会提出的各项任务，以“新北京、新奥运”为主题，以加快发展为总的基调，以改革创新为强大动力，以提高人民生活水平为根本出发点，实施科教兴国和可持续发展战略，保持首都经济持续快速健康发展，加快城市现代化建设和城乡一体化进程，促进社会主义物质文明、政治文明和精神文明协调发展，正确处理改革发展稳定的关系，在全面建设小康社会的基础上，努力率先基本实现现代化，为把北京建成现代化国际大都市奠定坚实的基础。

　　2003年，是全面贯彻党的十六大精神的第一年，又是新一届政府的届首之年。做好今年的各项工作，对于加速首都现代化进程，实现“新北京、新奥运”的战略构想，具有十分重要的意义。全市经济和社会发展的主要预期目标是：国内生产总值增长9%以上，地方财政收入增长15%左右；城镇居民人均可支配收入和农民人均纯收入实际增长6%以上；城镇登记失业人员就业率保持在60%以上，城镇登记失业率控制在2.5%以内；居民消费价格指数为101%左右。

　　(一)加强就业和社会保障工作，增加城乡居民收入，提高人民生活水平

　　就业是民生之本。扩大就业，增加收入，提高人民生活水平是政府工作的根本任务，要切实抓紧抓好，着力解决群众生活中的突出问题，使城乡居民生活得更方便、更舒心、更幸福。

　　突出抓好就业和再就业工作，促进城乡居民增收。要多渠道、多层次、多形式开发20万个以上的就业岗位，培育社区服务组织，鼓励并扶持发展劳动密集型产业、服务业、中小企业和非公有制经济，扩大就业门路。鼓励弹性就业、自谋职业和自主创业。高度重视高校毕业生的就业指导和服务。落实各项促进就业政策，启动“三年百万技能培训计划”，强化就业服务，改善创业环境。建立失业预警体系，对困难群体实施就业援助。要把增加农民收入与加强农村就业有机结合起来，拓宽劳务输出渠道，帮助5万农村富余劳动力实现就业。实施绿色生态富民工程纲要，全部消除年人均纯收入2500元以下的低收入村。理顺分配关系，完善和落实劳动、资本、技术和管理等生产要素按贡献参与分配的制度和机制。再分配要注重公平，强化公共财政职能，规范收入分配秩序，努力扩大中等收入者比重，提高低收入者收入水平，促进共同富裕。

　　深化社会保障制度改革，改善低收入群众生活。继续推进基本医疗保险制度改革，落实企业补充医疗保险，认真执行特困人员医疗救助办法，把农村特困居民纳入医疗救助范围，完善多层次医疗保险体系。力争自收自支事业单位全部参加养老保险。加快推进农转居人员参加社会保险的试点工作，扩大农村养老保险覆盖面。进一步加大扩面征缴力度，使各项社会保险基金收缴率达到95%以上。加强城乡最低生活保障的规范化和动态管理，符合条件的城乡低收入人员全部纳入最低生活保障范围。做好“两个确保”与“三条保障线”的衔接工作。建立市、区临时救助资金，构建最低生活保障、临时救助、生活补助和社会捐助相结合的社会救助模式，切实保障困难群众基本生活。

　　积极推进危旧房改造和住宅建设，办好60件实事。全年动迁居民5万户，拆除危房65万平方米。加大经济适用住房建设力度，力争全年竣工200万平方米，优先提供给旧城区危改和历史文化保护区拆迁居民认购。建设好廉租房小区，将具备条件的空置商品住宅转为廉租房屋，努力扩大廉租房源，解决更多双低家庭住房困难。继续办好60件实事，把群众在看病就医、社会治安、劳动就业、交通环保、物业管理等方面的突出问题解决好，使广大城乡居民在改革和发展中得到更多的实惠。

　　(二)坚持消费和投资双重拉动，发挥筹办奥运会的促进作用，推动首都经济持续快速健康发展

　　认真贯彻扩大内需的方针，逐步调整投资和消费的关系，把扩大消费摆到更加突出的位置，继续改善投资环境，优化投资结构，以奥运促发展，确保实现全年经济增长的预期目标。

　　促进潜在消费转化为现实消费，提高消费在国内生产总值中的比重。不断完善鼓励和引导消费的各项政策措施，加快个人信用制度建设，扩大消费信贷规模。推进银行卡工程，力争使持卡消费额占社会消费品零售额的比重提高到15%。积极推进住房分配货币化，房地产二三级市场的发展务必取得实质性进展。拓宽已购公房上市渠道，完善换房、租赁等住房消费政策，促进住房消费稳步增长。建成一批重点流通设施，加快发展连锁经营等新型商业业态，推进特色商业功能区建设。开发新的消费领域和空间，重点完善休闲消费等服务体系，促进服务性消费的快速增长，推动教育培训、旅游会展、文化体育、信息服务等消费向深层次发展。推进新型商业进农村，不断扩大农村市场规模。实现社会消费品零售额增长10%以上、力争达到2000亿元。

　　调整和优化投资结构，改善投资环境，降低投资成本。当前，投资仍是推动首都经济增长的主要力量。要充分发挥财政资金的引导和带动作用，广泛吸引社会投资和境外投资，全社会固定资产投资突破2000亿元。要以完善投资结构为主要着力点，拓宽资金来源渠道，发行好奥运建设企业债券，积极申请环保专项资金。搭建投资服务平台，研究控制工业用地价格的办法，降低在京投资特别是制造业投资成本，努力为各类投资主体创业和发展营造一流的环境。支持在京中央单位的建设与发展，吸引全国各类社会投资和外资参与首都现代化建设。引导社会资金的投向，把资金重点投向轨道交通和奥运场馆的建设，投向环境治理、危旧房改造，投向现代制造业、高新技术产业、现代服务业和传统产业的技术改造，投向郊区工业化和城市化建设，夯实经济增长的基础。

　　大力发展奥运经济，发挥奥运会对首都经济的加速器作用。奥运经济是首都经济发展在特定阶段的集中体现，筹办奥运会将大大促进消费和投资的增长。要认真实施《北京奥运行动规划》，制定实施“以奥运促发展”的具体措施，创新工作思路，加快发展步伐。抓住奥运会市场开发启动的有利契机，以拉动产业发展为目标，制定相关优惠政策，整合有实力的企业参与奥运赞助、供应和特许经营。加大奥运宣传，引导国内外企业全方位参与首都现代化建设，扩大奥运效应。积极实施奥运品牌战略，支持引导企业创世界级品牌和国内名牌，争取国内产品和技术在奥运会上应用推广。精心设计、宣传和经营城市，挖掘和丰富首都城市的独特魅力，着力培育城市品牌，塑造北京现代化国际大都市的形象。

　　(三)坚持首都经济的发展方向，加快产业结构调整，走新型工业化道路

　　坚定不移地走符合新型工业化道路要求、具有首都特点和时代特征的首都经济发展之路，壮大高新技术产业，振兴现代制造业，大力发展现代服务业和现代农业，在结构调整上迈出更大的步伐，实现经济增长速度与结构、质量、效益相统一。

　　推进中关村科技园区建设，培育高新技术产业群。落实《中关村科技园区五年上台阶行动纲要》，加快推进专业园和产业基地建设，软件园、上地北区等要初步具备企业大规模进驻的条件。建立和完善风险投资进入退出机制，建成中关村技术产权交易所。实施重点产业化项目，加速培育具有首都特色和竞争优势的高新技术产业群，逐步形成多元化发展的群体结构优势。继续把信息产业做大做强，抓住具有自主知识产权的关键技术，重点打造和延伸网络计算机、大规模图形图像专用芯片等产业链。高度重视软件产业的发展，重点支持一批具有相当规模的软件产业集团，扶持软件出口，加强软件人才培养。坚持以信息化带动工业化，加速“数字北京”工程建设，推进电子政务和电子商务，鼓励支持企业应用信息技术，保持首都信息化在全国的领先地位。

　　以汽车和微电子产业为龙头，进一步加大技术改造投入，振兴现代制造业。加速以顺义为中心的汽车生产基地建设，全力推进现代汽车合资项目，加快吉普车产品升级换代，推动北汽福田优化产品结构，尽快形成整车和零部件产品开发、生产销售、服务贸易为一体的产业发展格局。确保中芯国际集成电路生产线按期投产，加快京东方液晶显示器件项目建设，搞好集成电路设计园和国家集成电路研发中心建设，建成国内一流的集成电路产业基地，巩固发展北京微电子产业的领先地位。立足基地建设，促进生物工程和新医药、光机电一体化产业加快发展。推动高新技术和先进适用技术改造提升传统制造业，抓好北一大隈、燕化等一批重点项目。加快发展都市型工业。完成20家污染扰民企业的调整搬迁，压缩和淘汰落后生产能力。

　　大力发展现代服务业，促进第三产业优化升级。加快“三地一港”物流园区建设，全面启动招商引资，积极引进国际著名物流企业，整合现有物流资源，大力推行第三方物流，优化布局，构建现代流通业的基本框架。支持金融企业改革与发展，推进农村信用社改革，促进金融业务的扩展和创新。搞好商务中心区、金融街等重点功能区建设，吸引国内企业和各类金融机构来京投资金融产业。做好银行保险业利用外资试点准备工作，开展风险投资、基金运作等领域的对外合作。开发新型旅游方式和产品，树立北京旅游品牌，壮大旅游产业。扩大会展业规模。着力发展通信、网络、咨询等信息服务业。

　　统筹城乡协调发展，加速推进农业现代化、郊区工业化和城市化。按照增加农民收入，提高农业竞争力和可持续发展的要求，深化农业结构调整，切实做好退耕还林，大力发展都市型现代农业。推进农业产业化经营，重点扶持加工和贸易龙头企业，培育壮大优势主导产业，不断提高农业综合效益。促进农业标准化生产，全面实施“无公害食品行动计划”，深入开展农产品质量安全专项整治活动。搞好农村税费改革试点，推进集体资产产权制度改革，积极发展农产品行业协会和农民专业合作组织，加快农业科技推广体系和社会化服务体系建设。优化郊区产业布局，加大市级和区县工业开发区建设力度，适当整合规模较小的工业区，促进二、三产业快速发展，使郊区成为首都重要的产业基地和新的经济增长点。按照城乡一体化要求，加大基础设施和生态环境建设的投入，加快发展卫星城和具备条件的中心镇。完善农村土地征用制度，研究解决农业人口户籍、就业和社会保障等问题，切实保障农民利益，加快农村劳动力转移，推进农村城镇化。

　　(四)科学规划，加速建设，提升城市现代化管理水平

　　今年是奥运工程建设的启动之年，也是城市环境质量再上新台阶的关键一年。要全面实施“绿色奥运”战略，高标准规划、高质量建设、高效能管理、高水平经营城市。

　　切实提高城市规划设计和管理水平。修订《北京城市总体规划》，编制完成城市近期建设规划。改革规划编制体制，推行规划设计市场化运作，广泛引进国际国内一流规划设计力量，加快编制速度，提高编制水平。完善专家参与机制，提高规划决策的科学化和民主化。改革规划审批体制，实现由串联审批向并联审批转变，提高审批效率。依法加强规划监督管理，制止违法建设，查处违法建设行为。

　　着眼于从根本上缓解交通问题，实施300公里轨道交通建设规划。确保城市铁路东线和地铁八通线通车，加快地铁五号线工程实施和地铁四号线等新线开工建设。推进城市快速道路系统和城区路网加密工程建设，重点搞好交通节点的改造，建成五环路等重点道路，尽快缓解中心区交通压力。加快东直门、西直门等交通枢纽建设。坚持“公交优先”，扩大公交专用车道及公交优先车道，建成20条准快速公交线路，优化调整公共汽车、电车线网。推进出租汽车企业运营机制改革，建立良好经营秩序。坚持建管并举，加快建立智能化交通体系，加强静态交通管理，不断提高城市交通效率，使群众出行更加方便、快捷。

　　以启动奥运场馆建设为契机，高质量建设城市。加快奥运场馆项目法人招标、设计方案招标、建设用地拆迁平整等前期工作，确保奥林匹克公园市政配套和国家体育场、国家游泳中心等按期开工。务必做好重大工程建设的前期准备工作，开工建设国贸三期、第十水厂等一批新项目，推进国家大剧院、中央电视台、北京电视台、首都博物馆新馆等工程的建设。要加快电力设施建设和城网改造，着重抓好旧城平房区和新建集中居住区水、电、气、热、通信等设施的建设，进一步提高供给能力和服务水平。以南中轴路建设为契机，加大基础设施建设向南城倾斜力度，为加快南城发展提供条件。妥善处理好保护与发展的关系，搞好皇城的整体保护，修缮正觉寺等文物古迹，推进朝阜路、大栅栏等历史文化保护区整修试点。

　　以改善大气质量为重点，进一步加大环境治理和生态建设力度。采取更加严格有效的措施，力争市区空气质量二级和好于二级的天数达到60%。加大清洁能源使用比重，增加天然气供应能力，发展城市集中供热面积300万平方米，4个城区基本完成20吨以下燃煤锅炉改造。对新增机动车执行相当于欧洲2号排放标准，在用机动车排放达不到简易工况标准不允许上路。支持首钢完成调整和压产任务。削减市区工业用煤100万吨。加强道路交通和工地扬尘防治，基本完成城市裸露地面整治。继续实施水系治理工程。加快建设污水处理厂及中水回用工程，使市区污水处理率达到50%，中水回用日均达到24万吨。抓好园林、公共绿地节水设施改造，推广雨洪利用技术，加快建设节水型城市。加大固体废弃物、危险废弃物、噪声等污染的防治力度。搞好节能工作，提高资源利用率。加快以林业为重点的生态环境建设，基本完成第一道绿化隔离带125平方公里绿化和“五河十路”绿色通道建设任务，启动第二道绿化隔离带建设，加强湿地的恢复和保护工作。城市绿化要与改善人民群众生活条件、治理脏乱差、保护文物和美化环境相结合，每个区至少建设一处一公顷以上大绿地。继续治理城市中的脏乱点，完成100项重点环境整治项目，建设一批环境精品、亮点工程，展现北京更加亮丽的风采。

　　继续深化城市管理体制综合改革，全面推进社区建设。加强对城市管理工作的领导，进一步推动城市管理重心下移和事权下放，在城管、公安、工商、社保等工作进社区方面取得明显进展。加强社区自治组织建设，提高社区自我管理能力，强化社区服务功能，健全市、区、街相互衔接、合理分工和规范高效的城市管理框架，完善“统一领导、各司其职、规范管理、强化基层”的城市管理格局。推进行政处罚权相对集中，加大城市管理综合执法力度，建立社区城管监察执法“一区一员”联系人制度，全面实行责任监督，将严密到位的日常管理覆盖到全市各个方面，健全长效管理机制。

　　(五)优化发展环境，深化体制改革，扩大对外开放

　　首都经济的发展方向、核心和重点确立之后，改善和优化发展环境，已经成为当务之急。今年，要下决心把优化发展环境作为贯彻落实执政兴国第一要务的重大举措来抓，作为把握机遇的实际措施来抓，作为各级政府部门和工作人员改进作风、提高管理和服务水平的重要任务来抓。

　　不断改善和优化体制机制环境，深化国有资产管理体制和国有企业改革。按照十六大确定的国有资产管理体制改革方向和国家的法律法规，研究设立国有资产管理机构、改组调整国有资产授权经营公司的方案。打破部门和行业界限，重组、盘活经营性国有资产，实现国有资产保值增值。深化国有大型企业的股份制改造，着力完善法人治理结构，改进和加强监管机制。鼓励用国有企业的优良资产，吸引、嫁接、置换外资和民间资本，实现投资主体多元化，大力发展混合所有制经济。支持国内外大企业特别是跨国公司收购兼并国有企业。推进垄断行业改革，积极引入竞争机制。加快国有企业上市步伐，提高直接融资比重。推进高新技术企业股权激励试点。认真执行《中小企业促进法》，实施中小企业金融支持工程，进一步放开搞活国有中小企业。完善企业退出机制。

　　不断改善和优化公平竞争环境，推动非公有制经济发展壮大。毫不动摇地鼓励、支持和引导非公有制经济发展。凡是允许外资进入的领域，都向民间资本开放。私营企业在市场准入、上市融资、进出口、使用外汇、参与政府采购和招标投标、高新技术企业认定、申报政府计划项目、科技奖励、取得许可证和资质等级证书以及引进人才等方面，享受与国有、集体企业同等待遇。依法加强监督和管理，改善服务，尽一切可能使非公有制经济在首都经济社会发展和城市建设中发挥更为重要的作用。

　　不断改善和优化人才发展环境，发挥首都的人才优势。紧紧围绕首都经济和社会发展的目标及其对人才的总体要求，实施首都人才战略，抓住培养人才、吸引人才、用好人才三个关键环节，努力形成广纳群贤、人尽其才、充满活力的用人机制。开发利用国际国内人才资源，着力建设行政管理、企业经营和专业技术人才队伍。鼓励支持留学人员回国创业，广泛吸引国内优秀人才来京发展，承认和尊重外来务工人员的劳动贡献，发挥所有首都建设者的聪明才智，调动一切积极因素，为首都现代化建设服务。

　　不断改善和优化管理服务环境，提高政府工作效率。完善政府的经济调节、市场监管、社会管理和公共服务职能，努力实现经济增长、就业增加、物价稳定的经济调控目标。继续推进政府机构改革，完善公务员制度，切实转变作风，加大督查考核力度，提高办事效率和服务水平。坚决纠正行业和部门不正之风，坚持标本兼治，注重治本，从源头上预防和解决腐败问题。调整机构设置，理顺部门分工，进一步清理和精简行政审批事项，科学设定并规范审批程序，缩短审批时限。建立行政审批投诉制度和社会评价制度。大力培育各类行业协会，健全和规范社会中介组织，转移政府承担的社会职能。抓好中关村科技园区管理体制改革，吸收高新技术企业代表进入园区决策机构，使企业逐步成为园区管理的主体。完成有关规划、交通等管理体制改革方案的研究、实施工作。深化投融资体制改革，规范政府投资范围，改进政府投资项目管理，完善固定资产投资项目审批方式。强化部门预算改革，推进国库集中收付制度改革。集中财力办大事，提高财政资金使用效益。坚决制止铺张浪费，切实做好增收节支工作。建立全市企业信用信息系统和不良记录公示制度，加快建设市场经济的社会信用体系，改善信用环境。完善工程项目招投标、政府采购、经营性土地交易和国有资产产权交易等市场，健全现代市场体系，进一步整顿规范市场经济秩序，确保食品药品安全、金融安全和安全生产。优化结构，调控总量，保持房地产市场健康稳定发展。

　　不断改善和优化对外经贸环境，提高利用外资和外贸出口水平。认真实施《我国加入世界贸易组织过渡期北京行动计划纲要》，抓住外资大量进入我国的机遇，提高法规和政策透明度，全面优化投资环境，提高对外资的吸引力。逐步开放服务领域，扩大服务业利用外资规模。推广“星网工业园”引资经验，推动外资进入现代制造业，进入北京经济技术开发区等各类工业园区。继续吸引跨国公司在京设立地区总部、研发中心和采购中心。鼓励以存量引增量，扩大现有外商投资企业的投资规模。推进财政、税务、海关、商检、外汇管理等部门通力协作，巩固“大通关”成果。发挥进出口贸易预警机制的作用，维护产业安全。进一步完善落实各项出口政策，扩大高新技术产品和机电产品出口，提高服务贸易和技术贸易出口比重，力求实现出口市场、主体、产品和方式的多元化。解决企业对外投资中的项目申报和资金支持等问题，支持有比较优势的企业“走出去”，带动本市产品、技术出口和劳务人员输出。广泛开展国际交流与合作，多办并办好高水平的国际活动。深度参与西部大开发，进一步加强与兄弟省区市的交流与合作。

　　(六)大力发展社会主义文化，切实加强社会主义精神文明建设

　　强化首都文化中心功能，牢牢把握先进文化的前进方向，把精神文明建设摆在更加突出的位置抓紧抓好，努力建设学习型社会，使市民的思想道德素质、科学文化素质和健康素质有新的提高，为首都建设与发展提供强大的精神动力和智力支持。

　　积极推进教育改革和发展。坚持把教育放在优先发展的地位，适应首都经济社会发展需要，加大教育创新力度，深化教育教学改革，全面推进素质教育。发挥优质教育资源的辐射、带动作用，进一步提升各级各类教育的质量和水平。保证教育经费“三个增长”，提高使用效益。继续加大学校布局调整力度，大力改善办学条件，促进基础教育均衡发展。积极发展高中教育，扩大优质高中教育规模。加快良乡和沙河高教园区建设，稳步发展高等教育。大力加强职业教育、成人教育和社区教育，努力构建终身教育体系。注重师德教育，加强骨干教师队伍建设。学习贯彻《民办教育促进法》，鼓励社会力量以多种形式参与办学。扩大教育对外开放。

　　加快科技创新步伐。完善首都区域创新体系，继续抓好“首都二四八重大创新工程”，整合首都科技资源。大力发展科技服务体系，加强生产力促进中心建设。以专业孵化器和大学科技园为重点，完善创业孵化网络。继续深化科研机构转制，提高转制院所在市场环境下的生存和发展能力，加速科技成果向现实生产力转化。加强知识产权保护。普及科学知识，弘扬科学精神。坚持社会科学和自然科学并重，充分发挥哲学社会科学在经济和社会发展中的重要作用。

　　全面繁荣文化卫生体育事业。大力实施文化精品战略，努力创作一批叫得响、传得开、留得住的精品力作。积极扶持民族艺术，引进国外优秀文化艺术。支持和保障文化公益事业，建设一批文化设施，开展丰富多彩、健康向上的群众文化活动。深化文化体制改革，制定完善相关政策，广泛吸引各类资金、人才和企业进入文化产业，进一步把新闻出版、广播影视、文化娱乐等产业做大、做强。加大对文化市场的整治力度，坚持开展“扫黄打非”斗争，确保文化市场的繁荣、健康、有序。努力提高医疗质量和服务水平，加大公共卫生经费投入，健全疾病预防和急救系统，加快实施农村初级卫生保健发展纲要，逐步建立以大病统筹为主要形式的新型农村合作医疗制度，完善社区卫生保健功能，以低廉的价格为群众提供比较优质的服务。搞好计划生育工作，提供优质生殖保健服务，提高出生人口素质。广泛开展全民健身活动，继续配建“全民健身工程”，提升竞技体育水平，争办高水准的国际体育赛事，大力发展体育产业。高度重视和积极支持老龄工作，加快发展残疾人事业，切实保障妇女和未成年人的合法权益。

　　扎实推进首都精神文明建设。深入进行党的十六大精神和“三个代表”重要思想的宣传教育，切实把全市人民的积极性、创造性凝聚到率先基本实现现代化的奋斗目标上来。弘扬和培育以爱国主义为核心的民族精神，教育和引导全市人民树立中国特色社会主义共同理想，树立正确的世界观、人生观、价值观。切实开展好“首都公民道德实践年”活动，引导人们在遵守基本行为准则的基础上，追求更高的思想道德目标。广泛进行热爱祖国、热爱首都的群众思想教育，增强市民的首都意识和首善意识。加强国防教育，增强市民国防观念，高标准做好征兵和民兵预备役工作。广泛开展“双拥”共建活动，严格落实优抚安置政策，巩固和发展军政军民团结。深入开展文明区县、文明村(镇)、文明社区等创建活动。进一步做好乘车秩序、旅游环境、文明赛场等整治工作，下大气力治理随地吐痰、乱扔垃圾、随意张贴小广告等顽症。把市民讲外语活动引向深入。

　　(七)建设社会主义政治文明，确保首都安全稳定

　　坚持依法治国与以德治国相结合，扩大民主，健全法制，巩固和发展首都长期和谐稳定的良好局面，努力建设社会主义政治文明和精神文明的首善之区。

　　发展社会主义民主。各级政府要坚决贯彻人大及其常委会决议，坚持重大事项报告制度，自觉接受人大监督。加强与政协的联系，把政治协商纳入政府决策程序，为民主监督和参政议政创造良好条件。完善人民群众建议征集制度、与群众利益密切相关的重大事项社会公示制度和社会听证制度，政府的重大政策、重大规划和重大改革方案的制定都要有专家参与，促进决策民主化、科学化。扩大政府决策及实施的社会基础，保障群众的知情权和参与权。积极支持工会、共青团、妇联等群众团体开展工作。完善村民自治，推进社区自治，搞好社区居委会换届选举，扩大基层民主。全面贯彻党和国家的民族、宗教、侨务政策，开展民族团结进步创建活动，依法加强宗教事务管理，做好对台工作。

　　提高依法治市水平。加强行政立法，使立法工作不断适应首都改革发展稳定的要求。加强行政执法监督和行政复议工作，提高执法人员素质，提高依法行政的能力和水平。拓展和规范律师、公证、仲裁等法律服务，积极开展法律援助。加强法制宣传教育，将“四五”普法引向深入，认真学习宪法，树立宪法意识，维护宪法权威，增强全体市民的法律意识和法制观念，各级政府和领导干部要成为遵守宪法和法律的模范。

　　确保首都安全稳定。高度警惕、严密防范和坚决粉碎境内外敌对分子的各种颠覆破坏活动，突出反恐防暴，打击各类恐怖活动和民族分裂活动。坚持不懈地开展与“法轮功”邪教组织的斗争，坚决取缔、防范和依法打击各类邪教组织。坚持打防并举、预防为主，依法严厉打击各类犯罪活动，巩固严打斗争成果，强化社会治安综合治理，搞好基层安全创建活动，推进“科技创安”工程，加强社会面巡逻防控，提高社会整体防范能力和突发事件应变能力。狠抓安全生产责任制的落实，有效防止重大事故发生。做好信访、排查调处和人民调解工作，妥善处理好新时期人民内部矛盾纠纷，促进社会和谐稳定。

　　各位代表，本届政府任期已满，新一届政府即将产生。面对“新北京、新奥运”光荣而艰巨的任务，我们务必继续保持谦虚、谨慎、不骄、不躁的作风，务必继续保持艰苦奋斗的作风。牢记全心全意为人民服务的宗旨，密切同人民群众的血肉联系，廉洁奉公，为最广大人民的根本利益而不懈努力，不断把人民群众的利益维护好、实现好、发展好。

　　各位代表，中国共产党第十六次全国代表大会，掀开了我国社会主义现代化建设的新篇章，首都现代化建设在新世纪、新起点上，开启了一个新的历史征程。让我们在以胡锦涛同志为总书记的党中央领导下，高举邓小平理论伟大旗帜，全面贯彻“三个代表”重要思想，认真落实党的十六大和市第九次党代会精神，再接再厉，团结奋斗，与时俱进，开拓创新，加快建设现代化国际大都市的步伐，共同创造我们的幸福生活和美好未来！
